# Genomic prediction accuracies in space and time for height and wood density of Douglas-fir using exome capture as the genotyping platform

**DOI:** 10.1186/s12864-017-4258-5

**Published:** 2017-12-02

**Authors:** Frances R. Thistlethwaite, Blaise Ratcliffe, Jaroslav Klápště, Ilga Porth, Charles Chen, Michael U. Stoehr, Yousry A. El-Kassaby

**Affiliations:** 1 Department of Forest and Conservation Sciences, Faculty of Forestry, The University of British Columbia, 2424 Main Mall, Vancouver, BC V6T 1Z4 Canada; 2 Scion (New Zealand Forest Research Institute Ltd.), 49 Sala Street, Whakarewarewa, Rotorua, 3046 New Zealand; 3 Department of Genetics and Physiology of Forest Trees, Faculty of Forestry and Wood Sciences, Czech University of Life Sciences Prague, Kamycka 129, 165 21 Praha 6, Czech Republic; 4 Département des sciences du bois et de la forêt, Université Laval, QC, Québec G1V 0A6 Canada; 5 Department of Biochemistry and Molecular Biology, Oklahoma State University, Stillwater, OK 74078-3035 USA; 6 British Columbia Ministry of Forests, Lands and Natural Resource Operations, Victoria, BC V8W 9C2 Canada

**Keywords:** Douglas-fir, Genomic selection, Exome capture, Full-sib families, Genotype x environment interaction, Predictive model

## Abstract

**Background:**

Genomic selection (GS) can offer unprecedented gains, in terms of cost efficiency and generation turnover, to forest tree selective breeding; especially for late expressing and low heritability traits. Here, we used: 1) exome capture as a genotyping platform for 1372 Douglas-fir trees representing 37 full-sib families growing on three sites in British Columbia, Canada and 2) height growth and wood density (EBVs), and deregressed estimated breeding values (DEBVs) as phenotypes. Representing models with (EBVs) and without (DEBVs) pedigree structure. Ridge regression best linear unbiased predictor (RR-BLUP) and generalized ridge regression (GRR) were used to assess their predictive accuracies over space (within site, cross-sites, multi-site, and multi-site to single site) and time (age-age/ trait-trait).

**Results:**

The RR-BLUP and GRR models produced similar predictive accuracies across the studied traits. Within-site GS prediction accuracies with models trained on EBVs were high (RR-BLUP: 0.79–0.91 and GRR: 0.80–0.91), and were generally similar to the multi-site (RR-BLUP: 0.83–0.91, GRR: 0.83–0.91) and multi-site to single-site predictive accuracies (RR-BLUP: 0.79–0.92, GRR: 0.79–0.92). Cross-site predictions were surprisingly high, with predictive accuracies within a similar range (RR-BLUP: 0.79–0.92, GRR: 0.78–0.91). Height at 12 years was deemed the earliest acceptable age at which accurate predictions can be made concerning future height (age-age) and wood density (trait-trait). Using DEBVs reduced the accuracies of all cross-validation procedures dramatically, indicating that the models were tracking pedigree (family means), rather than marker-QTL LD.

**Conclusions:**

While GS models’ prediction accuracies were high, the main driving force was the pedigree tracking rather than LD. It is likely that many more markers are needed to increase the chance of capturing the LD between causal genes and markers.

## Background

Novel advancements in genomics technologies and statistical genetics, have paved the way for an increasingly prosperous environment for breeding industries. Notably in the dairy sector, the traditional phenotype-dependent selection has been successfully replaced by genotype-dependent selection (aka genomic selection, GS) which reduces the time required for evaluating genetic traits [1]. Tree selective breeding (aka tree improvement) challenges are somewhat similar to those of the livestock industry; namely, long generation times and low heritability and late expressing traits. GS, if successful, can offer unprecedented gains in forestry through reducing trait evaluation time, allowing faster breeding generations turn-over, and hence, an increased genetic gain per unit time can be reached. Additionally, the implementation of GS to forestry would offer a certain resilience to spontaneous market or environmental, and/or extraneous influences (e.g., disease/pest resistance, climate change); as breeding programmes adapt accordingly in a shorter time frame [2].

Selective breeding has traditionally focused on phenotypic selection, and more recently the linkage disequilibrium (LD) based indirect method of marker-assisted selection (MAS) [3]. MAS is suited for major-gene traits (such as resistance to white-pine blister rust (*Cronartium ribicola*)) and proven to be less effective for predicting complex quantitative traits that closely reflect Fisher’s infinitesimal model [4]. MAS models could not effectively describe a complex trait, since small effect loci are not readily discovered and considered ‘not significant’, thus leaving a substantial proportion of genetic control unaccounted for (i.e., missing heritability). Meuwissen et al. [5] proposed the method of ‘genomic selection’ to alleviate this problem by simultaneously considering all marker effects, and in doing so, all genetic contributions are captured regardless of size (significance). GS enables complex quantitative trait selection using genomic marker data alone and the method does not require a *priori* knowledge regarding the specific genetic architecture of the trait in question. Instead, markers throughout the entire genome (or in this case exome) are incorporated into the estimate. The resulting genomic estimated breeding values (GEBVs) for each individual derived from the GS models provide a basis, upon which selection decisions are made. The effect of this is a paradigm shift, in which the model unit of these breeding analyses shifts from being the ***line of decent*** to the ***allele***. This means that the phenotypic values of individuals are determined from genotypic data, enabling early selection of traits, leading to a significantly shorter breeding cycle and higher selection differential, particularly for the “difficult to assess” attributes.

The feasibility of applying GS to forest trees was initially assessed through deterministic simulations and the results indicated that GS has the potential to radically improve the efficiency of tree selective breeding [6]. Grattapaglia and Resende [6] also recommended further experimentation and proof of concept investigations. Since then, several forest tree species “proof-of-concept investigations” have been conducted with encouraging results [7–18].

Neves et al. [19] recognized that one of the major barriers to the application of genomic technologies to tree selective breeding was the large size of the genome of many forest species. This is particularly true of conifers, and although conifers have a large genome [20–22], their transcriptomes are comparable with other plants such as Arabidopsis whose genome is more than 100 times smaller [23]. Therefore, as an alternative to the prohibitive costs in both monetary terms and time, and the complexity of sequencing the whole genome, Neves et al. [19] focused on the coding region. This they refer to as “sequence capture”, and proposed that it would enable more efficient genetic variant identification in conifers; as it had previously been done in human and maize genomic studies [24–26]. Although sources of variation may not be exclusively found in the exome, the reduced cost and time compared to that of whole genome sequencing, as well as the ability to still capture a significant proportion of variants and rare variants, makes this method desirable as it is harder to find functional variants in non-coding regions [27, 28]. Exome capture has been recognized as an effective method for capturing rare variants in the field of medicine [29], and for increasing knowledge of unmapped large genomes [19, 28]. In addition, Suren et al. [20] have shown it to be a cost effective method for reducing complexity in large genomes, such as those of conifers.

The present GS study was based on the exomic information collected from 1372, 38-year-old coastal Douglas-fir (*Pseudotsuga menziesii* Mirb. (Franco)) trees. The samples represented 37 full-sib families with replications over three sites in coastal BC. The study objectives were: 1) to compare two GS methods; ridge regression best linear unbiased predictor (RR-BLUP) and generalised ridge regression (GRR), and 2) to test the GS prediction accuracy for within-, cross-, pooled multi-site, and time- time (age-age /trait-trait) between age 12 and 38 years. Two phases to this analysis were carried out, firstly the two GS models were trained on estimated breeding values (EBVs). This represents an analysis in which model predictions are based on pedigree (both historical and contemporary) and marker-QTL LD information. Secondly the models were trained on deregressed breeding values (DEBVs). In this analysis the pedigree information (parental average) is removed, resulting in model predictions based on marker-QTL LD and co-segregation. Results of ABLUP cross-validations are provided as a reference for comparison.

## Results

**Fig. 1:**
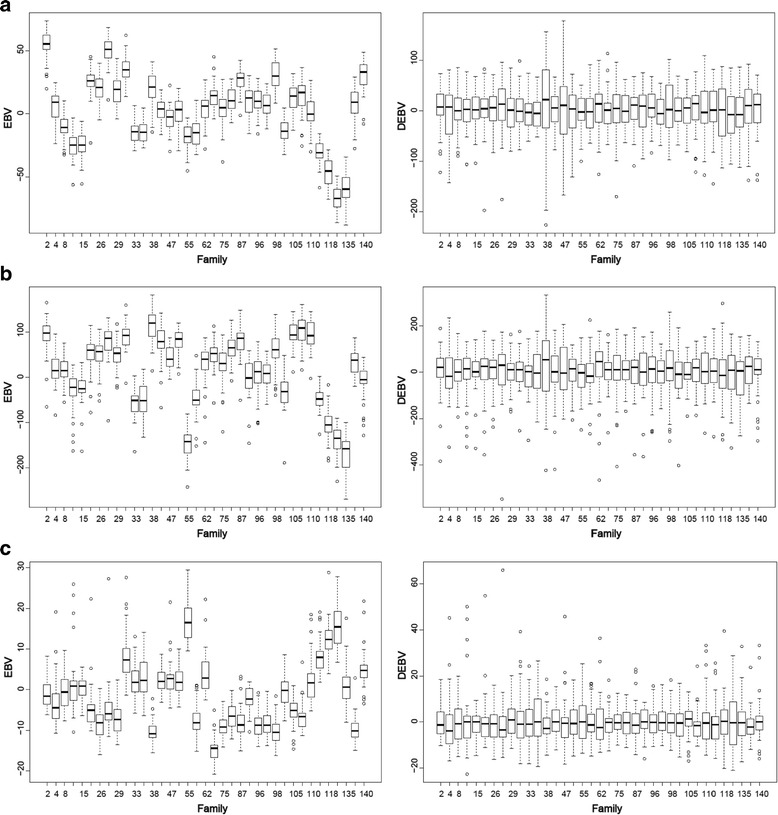
Distribution of estimated breeding values (EBVs) and deregressed estimated breeding values (DEBVs) for (**a**) height at 12 years (cm), (**b**) height at 35 years (cm), and (**c**) wood density (g/cm^3^) calculated from resistance to drilling

To assess the studied attributes’ variation, boxplots were produced showing the variance of the estimated (EBVs) and the deregressed (DEBVs) breeding values (Fig. 1). It is interesting to note that the deregression process maintained the within family variation for the three studied attributes (HT12, HT35, and WD_res_); however, it virtually eliminated among family variation resulting in similar family means (Fig. 1).

### Traits’ heritabilities and EBV accuracy

**Fig. 2:**
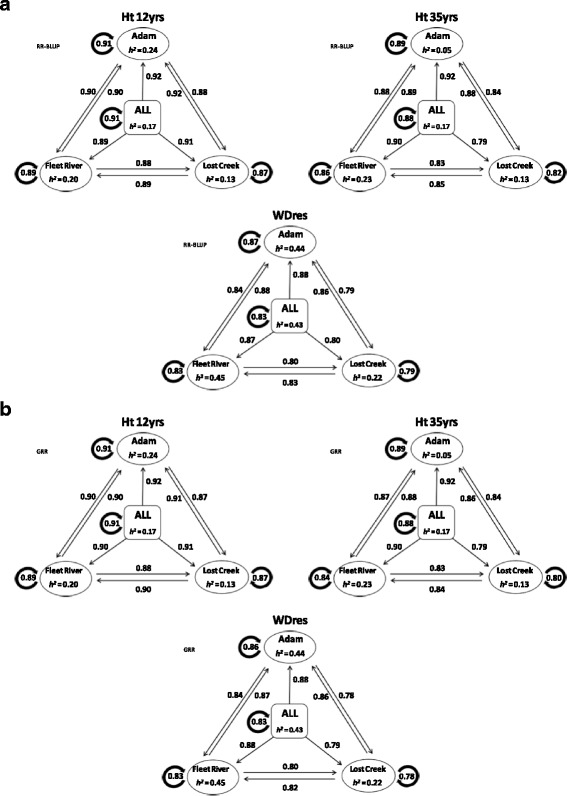
Heritabilities and GS prediction accuracies for models trained on EBVs and predicting GEBVs for each of the traits. Showing the results of within-site, cross-site, combined-sites, and multi-site to single-site cross-validation (Top (**a**), using RR-BLUP, Bottom (**b**), using GRR). The direction of the arrows depicts what information (site) is used to train the model (shaft end), and which is being predicted (head). Traits key: HT 35 yrs. = height at 35 years (cm); HT 12 yrs. = height at 12 years (cm); WD_res_ = wood density. Sites are Adam, Fleet River, Lost Creek, and multi/combined-site (ALL)

Pedigree-based relationship matrix (ABLUP) heights heritability estimates varied among sites ranging between 0.13 (Lost Creek) and 0.24 (Adam), and 0.05 (Adam) and 0.23 (Fleet) for age 12 and 35 years, respectively (Fig. 2). The multi-site height heritability estimates were similar to the average of the single-site estimates at age 12 (0.17 vs. 0.19); and was slightly higher than the average single site estimate by age 35 (0.17 vs. 0.14) (Fig. 2). Pedigree-based relationship wood density heritability estimates generally were higher than those obtained for height and substantially varied among sites (range: 0.22 (Lost Creek) and 0.45 (Fleet)), with higher multi- than single-site average estimates (0.43 vs. 0.37) (Fig. 2). The average theoretical accuracies for the EBVs were: 0.61, 0.68, and 0.76 for HT12, HT35, and WD_res_ respectively.

### Cross-validation across space and time

#### Within-site prediction accuracy

Within-site prediction accuracies, determined based on the correlations between EBVs and GEBVs for the two genomic selection (RR-BLUP and GRR) models, generally produced very similar results (correlations and standard errors) across EBV traits and sites (Table 1). For all EBV traits, Adam produced the highest prediction accuracies, with Fleet River second, and Lost Creek always producing the lowest accuracies. The two models, RR-BLUP and GRR, produced almost identical results in analysed EBV traits. The only differences occurring in the prediction of GEBV for HT35, in which the GRR method produced slightly lower prediction accuracies than RR-BLUP (0.84 vs. 0.86, and 0.80 vs. 0.82) for Fleet River and Lost Creek, respectively. The same was found in the analysis of WD_res_ for Adam (GRR = 0.86, RR-BLUP = 0.87). In contrast the WD_res_, GRR results for Lost Creek (0.84) were slightly more accurate than in the RR-BLUP model (0.79). It is worthy to mention that the overall predictive accuracy of the studied genomic selection models was generally high across sites and EBV traits (RR-BLUP: average = 0.86 and range of 0.79–0.91 and GRR: average = 0.86 and range of 0.80–0.91) (Table 1). Generally, all prediction accuracies’ standard error estimates were small reflecting good model fit.

**Table 1:** Within single-site prediction accuracies and their standard errors for ABLUP and genomic selection (ridge regression (RR-BLUP) and generalized ridge regression (GRR)) models for EBVs and GEBVs of heights (HT12 and HT35) and wood density (WD_res_)

Trait	Model	Site		
		Adam	Fleet River	Lost Creek
HT12	ABLUP	0.81 ± 0.002	0.77 ± 0.002	0.88 ± 0.001
	RR-BLUP	0.91 ± 0.010	0.89 ± 0.012	0.87 ± 0.013
	GRR	0.91 ± 0.010	0.89 ± 0.012	0.87 ± 0.012
HT35	ABLUP	0.85 ± 0.002	0.83 ± 0.002	0.88 ± 0.002
	RR-BLUP	0.89 ± 0.007	0.86 ± 0.020	0.82 ± 0.010
	GRR	0.89 ± 0.008	0.84 ± 0.020	0.80 ± 0.013
WD_res_	ABLUP	0.94 ± 0.001	0.96 ± 0.001	0.94 ± 0.001
	RR-BLUP	0.87 ± 0.007	0.83 ± 0.026	0.79 ± 0.015
	GRR	0.86 ± 0.008	0.83 ± 0.026	0.84 ± 0.016

Using the deregressed breeding values to train the two GS models, we obtained predictive accuracy results approximating 0.0 for WD_res_ for within-site cross-validation (RR-BLUP: Adam = −0.10 ± 0.055, Fleet River = −0.04 ± 0.046, and Lost Creek = −0.06 ± 0.049; GRR: Adam = −0.05 ± 0.074, Fleet River = −0.03 ± 0.045, and Lost Creek = −0.04 ± 0.046). The other models for HT12 and HT35 failed to converge.

**Fig. 3:**
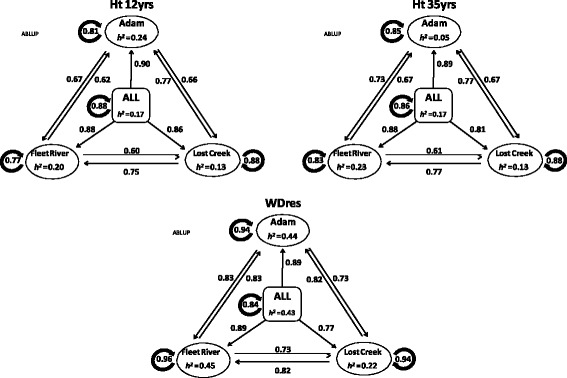
Heritabilities and prediction accuracies of the ABLUP model for each of the traits. Showing the results of within-site, cross-site, combined-sites, and multi-site to single-site cross-validation. The direction of the arrows depicts what information (site) is used to train the model (shaft end), and which is being predicted (head). Traits key: HT 35 yrs. = height at 35 years (cm); HT 12 yrs. = height at 12 years (cm); WD_res_ = wood density (g/cm^3^) calculated from resistance drilling to drilling. Sites are Adam, Fleet River, Lost Creek, and multi/combined-site (ALL)

The ABLUP within-site cross-validation, provided results of a similar nature as the GS models trained on EBVs. The average prediction accuracy for ABLUP within-site was 0.87 (both RR-BLUP and GRR had averages of 0.86), with a range of 0.77–0.96. WDres was predicted with the highest accuracy of all three traits, and surpassed the accuracy of the GS models: 0.94 ± 0.0009, 0.96 ± 0.0009, and 0.94 ± 0.001, for Adam, Fleet River, and Lost Creek, respectively (Fig. 3).

#### Cross-sites prediction accuracy

The average predictive accuracy of the RR-BLUP and GRR genomic selection models was very similar for cross- and within-site analyses. However, some trends in the predictive ability of the sites did occur (Fig. 2a and b). The sites Adam and Fleet River always produced the same or higher prediction accuracies for Lost Creek than the within site estimate for Lost Creek. In addition, Lost Creek cross-site prediction accuracies for Adam and Fleet River were also always higher than for itself. Fleet River always produced higher prediction accuracies for Adam than itself. This was true for all traits using EBVs. This may be an indication that whilst there may be some GxE occurring as expected, it may not be significant enough to employ single-site testing and breeding. The accuracy of cross-site predictions varied and ranged from 0.78 (GRR for WD_res_: Adam - Lost Creek) to 0.92 (RR-BLUP for HT12: Lost Creek - Adam,), and both selection models provided comparable results (Fig. 2a and b). Overall, across sites prediction accuracy decreased from HT12 to HT35 to WD_res_ with averages of 0.90, 0.86, and 0.83, respectively (all from RR-BLUP).

When the studied attributes’ deregressed breeding values (DEBVs) were used to train the two GS models, surprising outcomes were obtained and the resulting predictive accuracies were extremely low approximating 0.0 for WD_res_, while the remaining models for HT12 and HT35 failed to converge.

The results of the ABLUP cross-sites validation provided evidence of stronger GxE interaction than was predicted by the GS models (Fig. 3). This particular trend can occur as site-specific GxE interactions tend to over-estimate within-site prediction accuracy due to the sharing of a common environment, whilst seemingly inhibiting the efficacy of cross-site analyses. The average cross-sites prediction accuracies for ABLUP were as follows: 0.68, 0.70, and 0.79 for HT12, HT35, and WD_res_, respectively (Fig. 3). A drop from the accuracies obtained by the GS models trained on EBV data, and a complete reversal of the order of those accuracies.

#### Within multi-site (combined) prediction accuracy

The RR-BLUP and GRR models gave almost identical results, with HT12 having the highest combined-site prediction accuracy, followed by HT35 and lastly WD_res_ (0.91, 0.88, and 0.83, respectively for both RR-BLUP and GRR) (Fig. 2a and b, Table 2). In general, the combined site prediction accuracies were higher than the average of the within-site accuracies, except for WD_res_ which produced the same accuracy for both (RR-BLUP: 0.91 vs. 0.89, 0.88 vs. 0.86, 0.83 vs. 0.83; for HT12, HT35, and WD_res_, respectively) (Fig. 2a). Finally, it is also interesting to note the exceedingly small standard error values associated with all within multi-site prediction accuracies, highlighting the model(s) fit (Table 2).

**Table 2:** Within multi-site genomic selection prediction accuracies and their standard errors for ridge regression (RR-BLUP) and generalized ridge regression (GRR) models), estimating GEBVs and GEDBVs for heights (HT12 and HT35) and wood density (WD_res_) (g/cm3) calculated from resistance drilling to drilling

Trait	GS Model	GEBVs	GEDBVs
HT12	RR-BLUP	0.91 ± 0.004	−0.09 ± 0.019
	GRR	0.91 ± 0.003	−0.04 ± 0.017
HT35	RR-BLUP	0.88 ± 0.006	−0.02 ± 0.021
	GRR	0.88 ± 0.006	−0.01 ± 0.029
WD_res_	RR-BLUP	0.83 ± 0.009	0.00 ± 0.032
	GRR	0.83 ± 0.010	−0.01 ± 0.025

Again, using the deregressed breeding values (DEBVs) to train the two GS models, we obtained results approximating 0.0 for all within multi-site cross-validation analyses for all traits considered (HT12, HT35, and WD_res_) (Table 2).

The multi-site cross-validation accuracies for the ABLUP model were: 0.88 ± 0.002, 0.86 ± 0.003, and 0.84 ± 0.003, for HT12, HT35, and WD_res_, respectively (Fig. 3). Similar in magnitude and sequence to the GS models trained with EBVs.

#### Multi*-*site to single-site prediction accuracy

On average the multi- to single-site predictability for each trait was slightly higher than the within multi-site average estimates (for RR-BLUP, HT12: 0.91 vs. 0.89, HT35: 0.87 vs. 0.86, and WD_res_: 0.85 vs. 0.83) (Fig. 2a). In most cases the multi- to single-site accuracy predictions were the same or higher, than the corresponding single-site predictions. Except for HT35 at Lost Creek for both RR-BLUP and GRR which gave slightly lower prediction accuracies for multi- to single-site than within site (Lost Creek) analyses (RR-BLUP: 0.79 vs. 0.82; GRR: 0.79 vs. 0.80) (Fig. 2a and b). In most cases Adam was the most predictable site, and on average, Lost Creek was the least predictable.

Again, using the deregressed breeding values (DEBVs) to train the two GS models, we obtained results approximating 0.0 for all multi- to single-site cross-validation analyses.

The multi- to single-site predictions for the ABLUP model closely followed the pattern of predictions from the GS models trained on EBV data. In each case (for HT12, HT35, and WD_res_) Adam was the most predictable site (joint first with Fleet River for WD_res_), Fleet River the second, and Lost Creek the least predictable site (Fig. 3). The average multi- to single-site predictability for the traits were again the same or slightly higher than the within multi-site predictions for ABLUP (HT12; 0.88 vs. 0.88, HT35: 0.86 vs. 0.86, and WD_res_: 0.85 vs. 0.84) (Fig. 3).

#### Two sites predicting one site accuracy

**Fig. 4:**
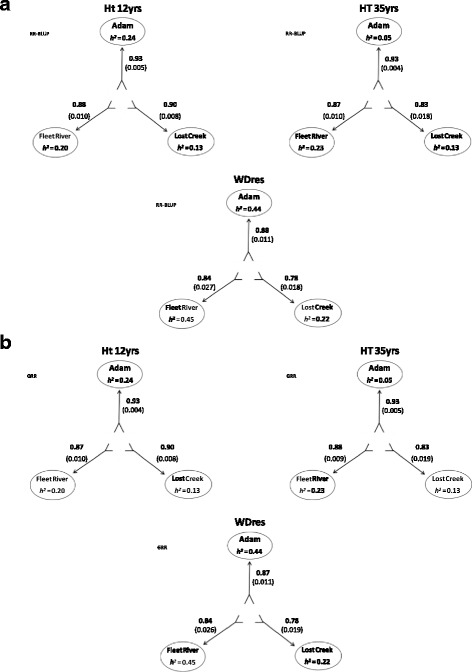
Heritabilities and GS prediction accuracies for models trained on EBVs and predicting GEBVs for each of the traits. Showing the results of two sites predicting one site cross-validation (Top (**a**) using RR-BLUP, Bottom (**b**), using GRR). The direction of the arrows depicts what information (site) is used to train the model (shaft end), and which is being predicted (head). Traits key: HT 35 yrs. = height at 35 years (cm); HT 12 yrs. = height at 12 years (cm); WD_res_ = wood density (g/cm^3^) calculated from resistance to drilling. Sites are Adam, Fleet River, Lost Creek

The RR-BLUP and GRR models for this analysis produced similar results overall (Fig. 4a and b). Generally, the highest prediction accuracies were obtained for HT12 (average = 0.90), followed by HT35 (average = 0.88), and lastly WD_res_ (average = 0.83) for both RR-BLUP and GRR. There were only minor differences between the two models (Fig. 4a and b). Similar to the multi- to single-site (above), the prediction accuracy of two sites to one site indicated that, in all cases, Adam was the most predictable site using this two to one analysis, despite the discrepancy in heritabilities, and on average Lost Creek was the least predictable in most cases.

The deregressed breeding values produced similar results to those obtained from within, cross- and multi-sites GS models, with two sites to one site cross-validation analyses prediction accuracy approximating 0.0 for HT12, HT35, and WD_res_.

**Fig. 5:**
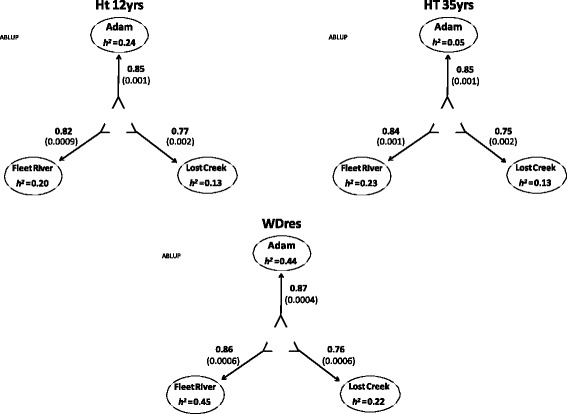
Heritabilities and prediction accuracies of the ABLUP model for each of the traits. Showing the results of two sites predicting one site cross-validation. The direction of the arrows depicts what information (site) is used to train the model (shaft end), and which is being predicted (head). Traits key: HT 35 yrs. = height at 35 years (cm); HT 12 yrs. = height at 12 years (cm); WDres = wood density (g/cm3) calculated from resistance to drilling. Sites are Adam, Fleet River, and Lost Creek

The ABLUP cross-validation for two sites predicting one site resembled the results of the GS model trained on EBVs. Prediction accuracies for HT12 and HT35 were lower than the GS models using EBVs (HT12 average = 0.81 vs. 0.90, and HT35 average = 0.81 vs. 0.88) (Fig. 5). However, the average predictability for WD_res_ remained the same for ABLUP as the GS models using EBVs (0.83) (Fig. 5). In general Adam was the most predictable site for all three traits (HT12, HT35 and WD_res_), followed by Fleet River and lastly Lost Creek in this ABLUP analysis (Fig. 5). This is the same site predictability trend displayed by the GS models trained on EBV data.

#### Time- time prediction accuracy (age- age/ trait-trait correlation)

In order to test the theory that the target time for forward selection can be reduced, prediction models were assessed on their accuracy when trained on younger trees (12 years: Ht_12_) followed by validation on the same trees for height at age 35 (Ht_35_) and wood density at age 38 (WD_res38)_ (i.e., correlations between GEBVs at age 12 and EBVs at ages 35 (HT_12_-HT_35_) and 38 (trait_HT12_-trait_WDres38_). The GEBVs values for Ht_12_ have significant positive correlations with EBVs for HT_35_ for both RR-BLUP (0.71 ± 0.0004) and GRR (0.71 ± 0.0004) (Table 3). Trait- trait correlation between HT_12_ and WD_res_ (recorded at 38 years: trait_HT12_-trait_WDres38_) produced significant negative correlations (RR-BLUP: -0.46 ± 0.0005; GRR: -0.46 ± 0.0006) (Table 3). These are the most useful correlations to make as they reflect the direction in which selections will be made.

**Table 3:** Genomic selection prediction accuracies for time- time correlations for HT12 for RR-BLUP and GRR models (standard errors)

GS Model	EBVs		DEBVs	
	WD_res_	HT35	WD_res_	HT35
RR-BLUP	−0.46 ± 0.0005	0.71 ± 0.0004	0.02 ± 0.0038	−0.03 ± 0.0039
GRR	−0.46 ± 0.0006	0.71 ± 0.0004	0.002 ± 0.0040	−0.004 ± 0.0047

Prediction accuracies based on correlations between GEBVs at age 12 and EBVs at age 35 (HT) and 38 (WD); and correlations between GEDBVs at age 12 and DEBVs at age 35 (HT) and 38 (WD). Traits key: HT35 = height at 35 years (cm); WDres = wood density (g/cm3) calculated from resistance drilling to drilling; HT12 = height at 12 years (cm)

Using the deregressed breeding values to train the two GS models, we obtained results approximating 0.0 for all time-time cross-validation analyses (Table 3).

Time-time and trait-trait cross-validation of ABLUP resembles closely that of the GS models trained on EBV data. EBVs for HT_12_ have a medium to strong positive correlation with EBVs at HT_35_ (0.74 ± 0.001), and a significant negative correlation with EBVs of wood density WD_res_ at age 38 (trait_HT12_-trait_WDres38_: −0.48 ± 0.002).

## Discussion

### Exome capture

Exome capture is a target enrichment method for sequencing the protein coding regions in a genome. This makes analysis much more efficient. Through targeting this region, the focus is immediately resolved to those areas that are likely to contain sources of variation for the phenotype. The reduced cost and time compared to that of whole genome sequencing, as well as the ability to still capture a significant proportion of variants makes exome capture desirable as it is harder to find functional variants in noncoding regions [20, 27, 28]. Added to which, forest trees already tend to have large and complex genome sizes [20–22]. Exome capture has been recognized as an effective method for increasing knowledge of unmapped large genomes [19, 28].

Whilst exome capture is expected to produce some missing marker information, the method surpasses whole genome shotgun sequencing or genotyping methods using genome complexity reduction, in depth of coverage. Given a restricted budget (with no option for re- sequencing) the depth achieved using exome sequencing is expected to be much greater than genotyping-by-sequencing [30], with the added benefit of obtaining more reliable calls. After the sequencing process, those markers with a large proportion of missing information can be filtered out, however Rutkoski et al. [31] admit that this step is unnecessary since it affects the GS prediction accuracy little. In order to maintain power in the subsequent analyses, marker imputation should be used to infer missing data in those records not filtered out, conserving the majority of the original SNPs. This was carried out in the current study, although the ratio of missing data was very low and therefore the impact of this procedure is expected to be minimal. Given that currently there is no genetic map available for Douglas-fir, imputation methods in this case are restricted to those which support unordered data. There have been various imputation methods proposed for unordered markers however their efficacy and suitability is yet to be fully determined in genomic selection [31]. Though some methods do show some promising results when compared to mean imputation, notably random forest regression produced greater GS prediction accuracy than its counterparts in both Rutkoski et al. [31] and Poland et al. [32] studies.

Although we managed to capture significant family effects using this type of SNP data, we found little evidence that the GS models were able to capture marker-QTL LD using this genotypic data set (see below for discussion). It is highly likely that substantially more SNPs will be required to capture significant effects for these traits (i.e., LD). In addition to this, our genotyping efforts were focused on a restricted portion of the genome, the exome, which is limited to the available 40 K probes used in the present study. In humans, the exome constitutes a mere 1% of the total genome [33], thus considering the unique genome size and complexity of conifers [34] we expect that the population of SNPs used in this study represents a very small fraction of the Douglas-fir genome. Additionally, in this case the revealed exome which represents functional genes that, by default, are under selection and thus are conserved. By focusing the present study on this region we were not able to capture the variation among families, as this was not represented in the exome. In order to seize this variation in intergenic regions, an alternative, whole genome approach must be used for genotyping, for example genotyping-by-sequencing as it uncovers unordered SNPs across the entire genome. These intergenic regions are not under the same selection pressure as the exome, and may contain important regulatory sequences which correspond to the control of the traits we investigated. Another approach used by Fuentes-Utrilla et al. [16], was to use restriction-site associated DNA sequencing (RADseq) technology. They found that in a species with no whole genome assembly (in this case Sitka spruce (*Picea sitchensis* (Bong.) Carr)), a SNP panel could be constructed from randomly located markers generated from RADseq. However, it must be noted that their GS analysis was performed strictly within a single family.

### Heritability

The use of ABLUP instead of GBLUP is likely falsely inflating the heritability estimate. Though the impact of heritability on the predictive accuracy seems to be low in this study. Our results show that even with modest heritability, predictive accuracies can be high. As shown by the combined site analyses, the heritability for HT12, HT35, and wood density were modest (0.17, 0.17, and 0.43, respectively), yet the prediction accuracies were 0.91 (± 0.004), 0.88 (± 0.006), and 0.83 (± 0.009), respectively (RR-BLUP) (Table 2). The large sample size and low effective population size of the present study (N_e_ = 21) likely helped in negating the effect of low trait heritability [5]. Mӓrtens et al. [35] provided evidence that increased relatedness between training and validation populations leads to higher prediction accuracy in their study on yeast. Furthermore, the use of correlation between pedigree-based and marker-based breeding values, approximates correlation between unknown true breeding value and genomic breeding value. The accuracy can go above heritability in this case because both values are representing only genetic effects, this is in line with results obtained by Gamal El-Dien et al. [12].

### Genomic selection

The desired outcome of genomic selection is to produce unbiased marker effect estimates [5], and to avoid the Beavis effect [36] which hinders MAS causing marker effects to be overestimated [37]. Instead of selecting markers based on a significance threshold, GS estimates all marker effects simultaneously causing a different problem; there are more predictor effects (*p*, markers in this case) to be estimated than there are observations (*n*, samples) [3]. Least squares cannot be used to estimate all the effects at once since there are not enough degrees of freedom. In addition, multi-collinearity of markers would cause any model to be over fitted [3].

To address this issue (large *p*, small *n*), various statistical models have been proposed. They generally fall into the following categories: shrinkage models, variable selection models, and kernel methods. Shrinkage models (e.g., ridge regression BLUP [RR-BLUP], Whittaker et al. [38]) fit all marker effects which are all shrunk to the same degree. With RR-BLUP, it is assumed that the trait in question more closely resembles Fisher’s infinitesimal model (many loci with small effects), and marker effects are samples from a normal distribution (with equal variance). Variable selection (e.g., generalized ridge-regression [GRR], Shen et al. [39]) however, reduces the number of markers used, and in doing so assumes that the trait is controlled by fewer, strong effect loci [3]. Kernel methods convert the predictor variables to distances in effect creating a matrix similar to an additive genetic relationship matrix. The kernel matrix quantifies the distance between individuals but also smoothing parameters are added [3]. This method is flexible and can incorporate complex relationships between markers, for this reason it is useful in cases where non-additive effects are suspected to occur [40]. Indeed, Douglas-fir height and wood density have proven to have non-additive genetic variance component [41]; however, its unpredictability has driven the species’ advanced generation breeding and selection methods to be additive genetic gain-dependent [41–43]. Since different traits have differing genetic architecture, there does not exist one model that is necessarily the best for all traits or populations [3].

In the present study, we assessed the prediction accuracies of two GS models (RR-BLUP and GRR) in two phases; first when trained on EBVs, and second when trained on DEBVs. In the first instance, using EBVs and by virtue of retaining family means, our data contained both contemporary and historical pedigree information as well as marker-QTL LD information. Without further adjustment, all this information was parsed into the GS models and resulted in high prediction accuracies (for both model types). Subsequent to this, in phase two, we deregressed the EBVs, removing the parent average effect in order to disassociate the pedigree information from the marker-QTL LD. This resulting in DEBVs that contained the marker-QTL LD information only without the between family genetic variance. Using these DEBVs to train the GS models, we obtained prediction accuracies which for all practical purposes were 0.0. The success of the first phase can be attributed to the large within and among family genetic variances. This pedigree-driven approach dominated the analysis and produced the observed high prediction accuracies, which were comparable to the ABLUP accuracies. When this influence of pedigree was removed, the generated models were no longer able to provide useful predictions. A similar effect was seen in interior spruce (*Picea glauca* x *Picea engelmannii*) where cross-validation using family folding resulted in decreased prediction accuracy, because between family variance was dominating the analysis (Gamal El-Dien et al. 2017, unpublished). In addition to this Fuentes-Utrilla et al. [16], when studying GS in Sitka spruce, found that within family predictions cannot be extrapolated to between families. Furthermore, when examining marker transferability between families in white spruce, Beaulieu et al. [44] also found within family predictions to be more precise than between family predictions. Similarly, to Gamal El-Dien et al. (2017, unpublished) they also found that when a family had no representation in the training group, the accuracies obtained for that family were very small, occasionally negative, and often not statistically significant from zero. Much like using the DEBVs here, which try to predict between family variance, but have family means stripped away. Fuentes-Utrilla et al. [16] concluded that species with large effective population sizes (notably conifers), have a reduced ability to make predictions across families. With this in mind, GS may best be employed to produce GEBVs for within large full-sib families in conifers as it captures the Mendelian sampling term.

This discrepancy in the results from the two GS phases, is indicative that we simply were not able to capture the marker-QTL LD with the available SNPs. Whilst other studies have had some success in this area, it is important to note that these particular investigations have focused on tree species with much smaller genomes, for example Resende et al. [7]. Resende et al. [17] make this point in their 2017 study of *Eucalyptus,* that although other studies may have failed to produce significant predictions between unrelated populations, this may be due to low marker density. This is likely inhibiting the ability to capture short-range LD, the result being that prediction models rely almost entirely on relatedness.

In their study, Resende et al. [7] used 7680 DArT markers on *Eucalyptus* which is estimated to have a genome size of 609 Mbp [7], equivalent to 12.61 markers/Mbp. In contrast, here we used 69,951 SNP markers on the Douglas-fir genome which is estimated at 18,700 Mbp, giving 3.74 markers/Mbp. To obtain a similar coverage as Resende et al. [7] would require approximately 235,800 markers, many more than we currently have. With such a large genome, it is likely that many more SNPs will be required (≈235,800+), with greater genomic coverage in order to capture this, so far elusive, LD. In a more recent study, also using *Eucalyptus,* Müller et al. [18] managed to capture some short-range historical LD using 5000–10,000 SNPs. Yet they too concluded that the genomic prediction in this case was largely driven by relatedness.

The similar predictions given by the RR-BLUP and GRR methods, and the similarly high ABLUP accuracies, was again the result on heavy reliance on between family variance, and thus we have gained no new information as to the genetic control or architecture of the traits in question. Although similar findings have been noted in more successful GS studies. For example, Resende et al. [8] when comparing GS methods, found there to be little difference between the predictive abilities of shrinkage and variable selection methods (4 in total) even considering they have different a *priori* assumptions. They used a *Pinus taeda* training population with 951 individuals and 4853 SNPs in the analyses. Prediction accuracies for 17 traits (including growth, disease resistance and development) ranged from 0.17 to 0.51. Only one trait (fusiform rust resistance) showed any significant difference between the models. Higher prediction accuracies were obtained using variable selection methods for this trait. This reflects the genetic architecture of the trait which is controlled by few, large effect loci [8].

### Single and multi-site cross-validation

The combined site GS analysis produced higher prediction accuracies than the single site analyses on average (Fig. 2a and b). The combined site training population having more individuals than the single site populations. This is in agreement with what the literature states should happen; Grattapaglia [2] noted that increasing the training population size increases accuracy up to a point (around 1000 individuals). In addition to increased sample size for predictive accuracy improvement, the multi-site approach incorporated the present GxE in the model, resulting in further improvement.

The prediction accuracies are high for HT12, HT35, and WD_res_ GEBVs (0.87–0.92, 0.79–0.92, and 0.78–0.88, respectively) (Fig. 2a and b). They are moderately higher than those in previous studies including other forest tree species [7, 8, 10, 11, 15]. Largely due to the inclusion of the pedigree structure. In this case the GS methods are not giving much advantage over ABLUP (ABLUP multi-site cross-validation accuracies: 0.88 ± 0.002, 0.86 ± 0.003, and 0.84 ± 0.003, for HT12, HT35, and WD_res_ respectively), thus both are predicting only family means. The prediction accuracies for both the GS models and the ABLUP model are much higher than theoretical accuracy of the EBVs (0.61, 0.68, and 0.76; for HT12, HT35, and WD_res_, respectively), which indicates that both the EBVs and GEBVs are converging on the family means, and are far from the true breeding values.

The high prediction accuracies for the GEBVs may also partially be the result of using a relatively large training population, known to correlate with accuracy. In addition, there is an interacting effect of the relatively low effective population size (N_e_ = 21), and both these data characteristics increase the accuracy of predictions [3]. Although in this case the low effective population/family size also meant that the Mendelian sampling term could not be defined. Indeed, Gamal El-Dien et al. [12] using an interior spruce population with N_e_ = 93.8 (estimated assuming the OP families are unrelated and contributed equally to the experiment) had lower prediction accuracies than obtained here. But these in turn were vastly higher than results from a study with 214 open-pollinated white spruce (*Picea glauca*) families in Quebec with N_e_ = 622.5 [9]. Another component responsible for the increased accuracy of predictions, was the training of the models on EBVs rather than raw phenotypes [12].

Prediction accuracies fell dramatically for models trained on DEBVs, as marker-QTL LD could not be recovered using the available SNP data set, indicating that the SNP markers effectively tracked the pedigree.

### Cross-site validation

Similar prediction accuracies were observed in the cross-site compared to the single-site and combined-site GS analyses (with models trained on EBVs). Genotype x Environment interaction is an important consideration of forest tree selective breeding, more so than in animal breeding where individuals are considered to share a common environment [45–48]. Prediction accuracy for HT12 across sites was relatively high. An unexpected result given the fact that the heritability was only 0.17 (combined sites), and there is expected to be a competition effect at early stand development (i.e., strong environmental component). This is possibly an indication of the partially controlled experimental environment (not natural stands) in which the trees were growing. Given these experimental conditions, it is also conceivable that GxE effects are minimal. Which is reflected in the cross-site predictions (Fig. 2a and b), which are of a similar magnitude to the within-site prediction accuracies for all attributes.

### Time- time and trait- trait correlations

It is thought that forward selection in Douglas-fir should be carried out at a minimum of 17 years [49], when accurate predictions of phenotype at time of harvest can be measured. We tested the correlation of height at 12 years and height at 35 years (HT_12_-HT_35_), and wood density (38 years) (trait_HT12_-trait_WDres38_) and positive (0.71 for height) and negative (−0.46 for wood density) correlations were detected by the GS models trained on EBV data. Although they did not offer accuracy above that of ABLUP, indicative of a strong reliance on pedigree information rather than marker-QTL LD. The results give an indication of how useful early selection could be. Correlations for height are moderately strong and low-moderate for wood density. Marker-trait associations are known to vary according to the tree age, limiting any correlation. This would certainly hamper efforts to create a highly correlated age-age model in trees [9, 50]. At the moment providing such predictions at an age any younger than 12 years would not be recommended (note that height at age 12 was the earliest measurement available in the present study). Since larger age differences have been shown to produce less accurate models [51]. The discrepancy in prediction accuracy between the time-time correlations and the cross-validations suggest that there is some inconsistency between EBVs and marker effects at these two ages (12 and 35 years). Although the discrepancy is relatively small, and so meaningful results may still be obtained through age-age and trait-trait correlations. This is, in addition to the varied environmental conditions the trees endure over their long lifespans, lessening time- time correlations. To this effect and as White et al. [52] suggested, training models will need to be updated with mid-rotation phenotypes in order to accurately predict mature trait values.

### Genomic selection and forest tree breeding

Several genomic selection “proof-of-concept” studies have been conducted on few coniferous tree species (e.g., loblolly pine, maritime pine, Sitka spruce, white spruce, and white-Engelmann spruce hybrid), all concluded that GS has the potential to increase the genetic gain through speeding breeding generation turn-over and increasing the selection differential. It should be stated that, with the exception of the maritime pine study [14], none of the derived GS predictive models have been validated on independent “validation” populations. The success of GS is dependent on the linkage disequilibrium between the markers used (i.e., SNP panels) and causal genes underpinning the traits of interest, and the degree of relatedness between the training and validation populations. Therefore, caution is required during GS implementation as LD changes after every round of breeding (i.e., recombination); the fact that it does rapidly decay called for using dense marker coverage to overcome this caveat. Still we have found that by only using sequence capture data, we were unable to successfully resolve this marker-QTL LD. We only had success in capturing among family effects (i.e., pedigree). Even with a SNP panel designed appropriately based on an informative SNP library, and large enough to handle a conifer genome, there are additional hurdles. LD only survives over relatively short distances in conifers compared to livestock species due to their relatively large N_e_ [53]. This led Fuentes-Utrilla et al. [16] to conclude that GS may only be useful in tree populations with reduced N_e_, for example seed orchards, or lines/ sub-groups which have been produced through selective breeding. Though as they demonstrate with their analysis, it is possible to generate very large full-sib families in trees, by controlled crossing. In this type of population, LD extends over longer distances than in open-pollinated populations. As a result, they suggest that GS could be employed to make selections within families.

Based on comparable studies: 1) a greater number of markers, and 2) wider coverage throughout the whole genome or dense unordered SNP genotyping platform (e.g., GBS), would be needed to capture this LD and additional intergenic variation [17]. Or indeed a shift in the level at which GS is applied, i.e. to within families rather than across families [16]. Whilst GS still has the potential to deliver unprecedented gains, it does not seem likely that was achieved in the present study as the prediction driving force was the pedigree rather than LD. Despite the low N_e_, the small family size prevented the accurate assessment of the Mendelian sampling term in this population. Therefore, the EBVs were heavily shrunk toward the family mean and all within family deviations were not estimated precisely.

Finally, it should be emphasized that any gains captured through GS require unique tree improvement delivery methods as the traditional seed orchards’ production mode requires time for reaching sexual maturity, even under intensive management such as top grafting or hormonal applications, and its sexual-production effectively breaks the LD between selected traits and markers.

## Conclusions

The results suggest that the population of SNP markers used, along with their low coverage across the Douglas-fir genome was not successful for capturing the LD with the causal genes underpinning the studied attributes. In this case the impressive results of the investigated genomic selection models relied heavily on relatedness rather than the LD. Alternative marker generation methods such as whole genome sequencing or other dense unordered SNP genotyping methods such as GBS are needed, as is a larger SNP array. Exome capture provide enough markers to successfully capture/track the pedigree (contemporary and historical) and thus it is useful for genetic variance decomposition of conifer traits, thus providing better genetic parameter estimates. Since we were only able to resolve the between family effects, we gained no new information regarding the genetic architecture of the traits. Whilst low N_e_ may help boost prediction accuracy in similar studies, there may be a lower limit to this. Beyond which Mendelian sampling is not captured. However, using a single, large full-sib family causes LD to extend over further distances compared to open-pollinated trees, therefore it is possible to make within family selections using this type of population as Fuentes-Utrilla et al. [16] have demonstrated.

## Methods

### Experimental population

Predictive models were trained on a replicated 38-year-old pedigreed coastal Douglas-fir (*Pseudotsuga menziesii* Mirb. (Franco)) progeny testing population. The trial was established by the Ministry of Forests, Lands and Natural Resource Operations of British Columbia, Canada in 1975 and consists of 165 full-sib families (54 parents), of which 37 families were selected for sampling from three test sites (**Adams** (Lat. 50 24′42″ N, Long. 126 09′ 37″W, Elev. 576 mas), **Fleet River** (Lat. 48 39′25″ N, Long. 128 05′05″ W, Elev. 561 mas), and **Lost Creek** (Lat. 49 22′15″ N, Long. 122 14′07″ W, Elev. 424 mas)) with a total of 1372 trees (N ≈ 500 per site) and effective population size (N_e_) of 21.

### Tissue sampling, DNA extraction and genotyping

Cambial tissue was collected using a hammer and hollow punch tool (approx. 2 cm diameter) to remove two small circular pieces of bark/ cambium and developing tissue from each tree. The cambium disks were separated from the bark layer and immediately placed in a 2 ml collection tube with 1 ml storage buffer (10 mM EDTA pH 8.0, 10 mM Na_2_SO_3_) and kept at 4 °C until DNA extraction. DNA extraction followed the modified procedure developed by Ivanova et al. [54] (R. Whetten, unpublished, North Carolina State University, personal communications). Genotyping was done using the exome capture method in a commercial facility (RAPiD Genomics©, Florida, US). A total of 40 K probes were designed using the available Douglas-fir transcriptome assembly [55]. From the Douglas-fir reference transcriptome, a total of 325,372 non-overlapping 120-bp probes were initially designed. After filtering out redundant and organelle matching probes, this number was reduced to 117,135 probes. Of the remaining probes, we selected 7464 that contained 17,096 SNPs previously reported [55]. A further 32,536 probes were selected bringing the total to 40 K. Selection was performed by randomly sampling the remaining probes restricting the selection to a maximum of two probes per transcript. These 40 K (7464 + 32,536) probes cover a total of 21,187 transcripts. The raw sequenced reads were demultiplexed in each individual barcodes. Low quality bases with less than 20 quality score in the 3′ end were trimmed out followed by a low quality filter that removed reads with more than 10% of the read with less than 20 quality score. The filtered reads were aligned against the reference transcriptome using Mosaik v2.2.3 [56] with the following parameters -mmp 0.05 -m all -a all -hs 15. SNP markers were identified at a population level using Freebayes [57] without considering indels, multi-nucleotide polymorphisms and complex events. This analysis resulted in approximately 550,000 SNPs. These SNPs were filtered to identify the highest quality sites. These included only biallelic SNPs with less than 40% missing data. Further filtering was applied so that data was located on contigs with a mean read depth less than or equal to 60. Additional filters included minor allele frequency (MAF) >5%, Hardy-Weinberg disequilibrium cut-off <−0.05, and maximum site depth < 60. This process resulted in a total of 1372 samples with 69,551 SNPs for use in the study, and mean imputation was used for the missing data. (for more details on exome capture see, Neves et al. [19]).

### Phenotyping

Early- (1988) and mid- (2011) rotation growth trait measurements of the studied trees had been assessed for height (HT: in meters) and mid-rotation (age 38 years, 2014) wood density (WD_res_) was assessed indirectly using the average resistance measurements recorded with a Resistograph® (Instrumenta Mechanik Labor, Germany). Resistograph readings were subsequently converted to wood density indices (g/cm^3^) by scaling them by the DBH measurements, as performed by El-Kassaby et al. [58].

### Estimated breeding values (EBVs) and deregressed breeding values (DEBVs)

EBVs were fitted in ASReml 3.0 [59], using the following mixed linear model for a single site:

1 $$ y=\boldsymbol{X}\boldsymbol{\beta } \kern0.5em +\boldsymbol{Z}a+e $$

where ***y*** is the phenotypic trait measurement, ***β*** is a fixed effect vector (overall mean), ***a*** is a random effects vector (additive genetic) which are normally distributed (~N(0, ***A*** σ _a_^2^ )) where ***A*** is average numerator relationship matrix and σ _a_^2^ is additive genetic variance, ***e*** is the random residual effects which are normally distributed (~N(0, ***I*** σ _e_^2^ )) where ***I*** is identity matrix and σ _e_^2^ is residual variance. ***X*** and ***Z*** are incidence matrices relating the fixed and random effects to the observations. Since GxE plays an important role in forestry [60] the combined site EBVs were estimated with terms accounting for site, replication, and family structure. Thus minimising biases in BV calculation caused by environmental variations between and within sites, and full-sib genetic effects.

Narrow-sense heritability was calculated as *h* ^2^ = σ_a_^2^ / (σ_a_^2^ + σ_e_^2^), where σ_a_^2^ and σ_e_^2^ are the variances of additive genetic and residual effects, respectively. Combined site heritability estimates included the GxE model term in the denominator. The breeding values (*â*) are fitted using BLUP as follows:

2 $$ \widehat{\mathrm{a}}={AZ}^{'}\ {\upsigma}_a^2\kern0.5em {V}^{-1}\ \left(y-X\ \widehat{\beta}\right) $$

where ***V*** is the variance- covariance matrix of ***y*** obtained by:

3 $$ \boldsymbol{V}=\mathrm{Z}{AZ}^{'}\ {\upsigma}_a^2\kern0.5em +I\ {\upsigma}_e^2 $$

Breeding values were deregressed using the deregression procedure of Garrick et al. [61]. This adjusts the BV data to account for family means, resulting in DEBVs that contain information regarding individuals only, without parental BV influence.

The theoretical accuracy of the EBVs ($\widehat{r}$) was calculated following the procedure of Dutkowski et al. [62].

4 $$ \widehat{r}=\sqrt{1-\frac{SE_i^2}{\left(1+{F}_i\right){\widehat{\sigma}}_a^2}} $$

where *SE*_*i*_ is the standard error of breeding value, and *F*_*i*_ is the inbreeding coefficient of the *i* ^th^ individual.

### Cross-validation across time and space

The two GS models (RR-BLUP and GRR) were compared to assess their application to various traits, and in addition they were cross-validated in order to assess their prediction accuracy across environments/ spatial divisions and time. The intention was to give an indication to which extent (if any) GS increases gain per unit time over traditional methods.

The validation processes used was a replicated randomly assigned 10-fold cross validation. Models were trained on 9/10 of these folds, with the remaining 1/10 fold used as a validation set. Prediction accuracy was determined as the mean of the replications of the Pearson product-moment correlation between EBVs of the validation set and their predicted GEBVs. Or in the case where DEBVs were used to train the models, the correlation between DEBVs and predicted GEDBVs (genomic estimated deregressed breeding values). The following combinations were used: 1) within-site, using information from a single site to estimate the GEBVs within that same site, 2) Cross-site/ between sites in all combinations, with information from single sites used to predict other sites, 3) Combined sites, pooled information from all the sites, 4) Multi-site to single site, the pooled information from all three sites used in the estimation of single sites only, 5) Two sites to predict one site, pooled information from two sites used in the estimation of the remaining site only, and 6) Time-time (age-age)/ trait-trait, using information from individual trees to obtain correlations between GEBVs at age 12 and EBVs at ages 35 for height and 38 for wood density. In addition, the same 10-fold cross-validation process was used to assess the predictive accuracy of the ABLUP model for all the spatial cross-validation analyses for all the attributes (HT12, HT35, and WD_res_) in the study. In this case predictions made were based on relatedness as given by the pedigree.

### Genomic selection analysis

Two GS methods were compared: ridge regression (RR-BLUP) and generalized ridge regression (GRR) [3]. The performance of each of the methods was assessed according to their predictive accuracy determined by the correlation between GEBVs and EBVs, or DEBVs and GEDBVs.

### Genomic estimated breeding values (GEBVs) and genomic estimated deregressed breeding values (GEDBVs)

#### RR-BLUP

Ridge regression (RR-BLUP: Whittaker et al. [38]) was proposed for use as a selection tool based on marker information. The model was fitted using the R package ‘RRBLUP’ [63], the GEBV (or GEDBV if using deregressed BVs) is obtained by the sum of *p* marker effects:

5 $$ g\left({x}_i\right)={\sum}_{k=1}^p{x}_{ik}{\beta}_k $$

where *x*_*ik*_ is the score (genotype) of SNP *k* in individual *i* , β _*k*_ is the marker effect of *k.* This method assumes that the marker effects are normally distributed (mean = 0) and so the BLUP solutions for marker effects are derived from solving mixed linear model equations of Henderson [64] so that ʎ is optimised in the following Eq:

6 $$ \widehat{\beta}={\left({Z}^{\prime }Z+\lambda \mathbf{I}\right)}^{-1}\ {Z}^{\prime }y $$

where ***Z*** is an incidence matrix relating markers to individuals, ***I*** is an identity matrix, and ***y*** is the vector of EBVs fitted in ASReml. The shrinkage parameter (ʎ) can be written as ʎ = σ _e_^2^ /σ _β_^2^ (residual variance / common marker effect variance). Since marker effects are assumed to be identically distributed, all effects are shrunk equally towards zero. This method is equivalent to using lines (as opposed to markers) as random effects in a mixed model analysis, where the covariance is modelled by a kinship matrix calculated from the marker data (a genomic relationship matrix [ ***G*** matrix]) (this is sometimes referred to as GBLUP).

#### GRR

Generalized ridge regression (GRR) is a two-step variable selection method, the first step obtains estimates in the same way that RR-BLUP does using linear mixed model analysis to solve for optimum *ʎ*, and in the second step the BLUP for $\widehat{\beta}$ is subjected to an alternative shrinkage parameter which is marker specific, using GRR to solve a heterogeneous error model which replaces ***I*** *ʎ* in (5) with *diag(ʎ)*:

7 $$ \widehat{\beta}={\left({Z}^{\prime }Z+\mathit{\operatorname{diag}}\left(\lambda \right)\right)}^{-1}{Z}^{\prime }y $$

In this case *ʎ* is a vector of *p* shrinkage parameters. For the *k* ^*th*^ element: ${\lambda}_k=\widehat{\sigma_e^2}\kern0.5em /\widehat{\sigma_{\beta k}^2}\kern0.4em$, is the parameter, where $\widehat{\sigma_{\beta k}^2}\kern0.5em$ is the variance of marker effect *k* ($\widehat{\sigma_{\beta k}^2}\kern0.5em ={\widehat{\beta}}_k^2/\Big(1-{h}_{kk}$))*.* $\widehat{\beta}$ is from step 1 (the BLUP marker effect) and *h*_*kk*_ is the influence of the dependant variable on the fitted value for observation *k.* In other words, *h*_*kk*_ represents the diagonal element (*n + k*) of the influence matrix ***H*** *=* ***T(T’T)*** ^−*1*^ ***T’*** *,* and:

8 $$ T=\left(\begin{array}{cc}X& Z\\ {}0& \mathit{\operatorname{diag}}\left(\lambda \right)\end{array}\right) $$

## Abbreviations

ABLUP: Pedigree-based Best Linear Unbiased Predictor
DEBV: Deregressed estimated breeding value
EBV: Estimated breeding value
GBLUP: Genomics-based Best Linear Unbiased Predictor
GRR: Generalized Ridge Regression
GS: Genomic selection
GxE: Genotype-environment interaction
HT12: Height at age 12 years
LD: Linkage disequilibrium
MAS: Marker-assisted selection
QTL: Quantitative trait loci
RR-BLUP: Ridge Regression Best Linear Unbiased Predictor
WD_res_: Wood density estimated using a Resistograph
